# Evolution of *gag* and *gp41* in Patients Receiving Ritonavir-Boosted Protease Inhibitors

**DOI:** 10.1038/s41598-017-11893-8

**Published:** 2017-09-14

**Authors:** Justen Manasa, Vici Varghese, Sergei L. Kosakovsky Pond, Soo-Yon Rhee, Philip L. Tzou, W. Jeffrey Fessel, Karen S. Jang, Elizabeth White, Thorsteinn Rögnvaldsson, David A. Katzenstein, Robert W. Shafer

**Affiliations:** 1 Division of Infectious Diseases, Department of Medicine Stanford University, Stanford, CA USA; 2 Department of Biology, Temple University, Philadelphia, PA United States; 3 Department of Internal Medicine, Kaiser Permanente Medical Care Program - Northern California, San Francisco, CA United States; 4 School of Information Technology, Halmstad University, Halmstad, Sweden

## Abstract

Several groups have proposed that genotypic determinants in *gag* and the *gp41* cytoplasmic domain (*gp41*-CD) reduce protease inhibitor (PI) susceptibility without PI-resistance mutations in protease. However, no *gag* and *gp41*-CD mutations definitively responsible for reduced PI susceptibility have been identified in individuals with virological failure (VF) while receiving a boosted PI (PI/r)-containing regimen. To identify *gag* and *gp41* mutations under selective PI pressure, we sequenced *gag* and/or *gp41* in 61 individuals with VF on a PI/r (n = 40) or NNRTI (n = 20) containing regimen. We quantified nonsynonymous and synonymous changes in both genes and identified sites exhibiting signal for directional or diversifying selection. We also used published *gag* and *gp41* polymorphism data to highlight mutations displaying a high selection index, defined as changing from a conserved to an uncommon amino acid. Many amino acid mutations developed in *gag* and in *gp41-*CD in both the PI- and NNRTI-treated groups. However, in neither gene, were there discernable differences between the two groups in overall numbers of mutations, mutations displaying evidence of diversifying or directional selection, or mutations with a high selection index. If *gag* and/or *gp41* encode PI-resistance mutations, they may not be confined to consistent mutations at a few sites.

## Introduction

HIV-1 can develop resistance to protease inhibitors (PIs) as a result of protease mutations that reduce PI binding affinity or compensate for the reduced fitness associated with PI binding site mutations^1^. Such mutations occurred commonly when PIs were used without ritonavir-boosting (PI/r) or as part of incompletely suppressive antiretroviral (ARV) treatment regimens. However, protease mutations responsible for PI resistance are now uncommon in patients with virological failure (VF) on an initial PI/r-containing regimen, particularly those including lopinavir (LPV/r), atazanavir (ATV/r), or darunavir (DRV/r)^2–5^.

Two explanations have been proposed for this decrease in primary PI-resistance. The first explanation is that PI-resistance mutations in protease develop only in viruses exposed to a narrow window of suboptimal drug concentrations that both exert selective pressure on the virus and allow virus replication^6^. This explanation is supported by the observations that patients receiving PI-containing regimens are also at reduced risk of developing NRTI-resistance mutations^7, 8^, and that most patients with VF on an initial PI-containing regimen but without PI-resistance protease mutations achieve virological re-suppression with improved adherence alone^9–11^.

The second explanation is that mutations outside of protease reduce PI susceptibility even in the absence of PI resistance mutations in protease^12, 13^. These mutations may have previously eluded detection because standard genotypic resistance testing is limited to HIV-1 protease, RT, and integrase, and because the most commonly used phenotypic assays create recombinant viruses that assess only those genotypic determinants present in these three genes and the C-terminal region of *gag* ^14^. Indeed, many studies have shown that genetic changes in *gag* including protease cleavage site mutations and non-cleavage site mutations may influence PI susceptibility (reviewed in Fun *et al*.^12^). Additionally, the Rabi *et al*.^13^ study reported that pseudotyped viruses containing *gp41*-CD from PI-resistant strains can reduce PI susceptibility in the absence of PI-resistance protease mutations.

In this study, we sequenced HIV-1 *gag* and *gp41* before and after therapy with ATV/r or LPV/r to determine whether these genes contained evidence for PI/r selective pressure and whether there are specific *gag* and *gp41* mutations consistently arising in viruses from PI/r-experienced individuals upon confirmed VF. For comparison, we also sequenced paired viruses from a control group of individuals that received a non-nucleoside RT inhibitor (NNRTI)-containing regimen as first-line therapy and experienced VF.

## Methods

### Individuals and samples

The study subjects included HIV-1-infected individuals in the Kaiser Permanente Medical Care Program of Northern California (KPNC) who had genotypic resistance tests performed at Stanford University Hospital between April 2001 and June 2013, and participants in the ACTG A5202 clinical trial^8^. Each group of study subjects met the following criteria: (i) Received ATV/r or LPV/r but no other PI; (ii) Developed VF while receiving ATV/r or LPV/r; and (iii) had cryopreserved samples obtained before and after receiving PI therapy. In KPNC, VF was defined as having one or more plasma HIV-1 RNA levels ≥75 copies/ml while receiving therapy. In A5202, VF was defined as confirmed plasma HIV-1 RNA level ≥200 copies/ml after 24 weeks. The control group comprised individuals from KPNC and A5202 with VF on a first-line NNRTI-containing regimen. This study was approved by the Institutional Review Boards (IRBs) of Stanford University, KPNC, and the NIH ACTG and all study methods were performed in accordance with the guidelines of these IRBs.

### Amplification and sequencing of *gag* and *gp41*

Plasma samples were ultracentrifuged and the resulting pellet was subjected to RNA extraction and reverse transcription using a high-fidelity reverse transcriptase enzyme (Super Script III, Invitrogen, Carlsbad, CA, USA). Random primers were used for cDNA synthesis of *gp41* and gene-specific primers were used for *gag*. A 50 µl first-round PCR was carried out with Platinum Taq High Fidelity DNA polymerase (ThermoFisher Scientific, Fremont, CA, USA) using first round primer pairs 622F and 2621R for *gag* and ES8FWD and CAM9038 for *gp41* (Table 1).

**Table 1:** Primers Used for Nested PCR Amplification of gag and gp41.

Gene	Primer name	Primer	Direction	HXB2 Numbering
gag	622F	AAAATCTCTAGCAGTGGCGCCCGAACAG	Sense	622 → 649
	685F	CTCGACGCAGGACTCGGCTTGCTG	Sense	685 → 708
	2577R	CTGGTACAGTTTCAATAGGACTAATGGG	Antisense	2550 ← 2577
	2621R	CCATTGTTTAACTYTTGGVCCATCCATTCC	Antisense	2592 ← 2621
gp41	ES8FWD	TGAGGGACAATTGGAGAAGTG	Sense	7648 → 7668
	7779	TTCCTTGGGTTCTTGGGAGCAGCAGG	Sense	7779 → 7804
	CAM9024	TAAAGGTACCTGAGGTNTGACTGG	Antisense	9001 ← 9024
	CAM9034	TCATTGGTCTTAAAGGTACCTGAGG	Antisense	9010 ← 9034

A 100 µl second-round PCR was carried out with primers 685F and 2577R for *gag* and 7779F and CAM9024 for *gp41*. The PCR cycling conditions were 94 °C for 120 seconds, followed by 30 cycles of (94 °C for 15 seconds, 55 °C for 20 seconds, 72 °C for 180 seconds), followed by a final extension at 72 °C for 10 minutes. The second-round PCR amplicons encompassed codons 45 to 345 of the 345 *gp41* codons and codons 1 to 500 of the 500 *gag* codons. PCR products were purified prior to dideoxyterminator Sanger sequencing. The 40 pairs of *gag* and 45 pairs of *gp41* sequences have been submitted to GenBank under the following accession numbers: KT33954 to KT340052, and KY579814 to KY579947.

### Analysis

To identify gene-wide evidence of selection pressure in *gag* and *gp41*, we performed two analyses: (i) We obtained maximum likelihood estimates of dN/dS – the ratio of non-synonymous and synonymous substitution rates – for each pair of sequences obtained at baseline and follow-up for the PI-treated and control NNRTI-treated individuals; and (ii) We used the RELAX test, as implemented in HyPhy v2.3^15^, to detect shifts of selective pressure using codon-substitution phylogenetic models^16^. RELAX compares the relative strength of selection between two sets of branches (in our case, the pre- and post-therapy branches) and expresses it as a single value, *K*, with *K* < 1 (with a significant likelihood ratio test p-value versus the null of *K* equals 1) indicating a weaker selective pressure on the post-therapy branches, relative to the pre-therapy branches.

To identify evidence for selection pressure at individual amino acid positions, we performed three analyses: (i) We ran the fixed effects likelihood (FEL) method, as implemented in HyPhy v2.3, to detect codon sites exhibiting diversifying selection in *gag* and *gp41* using a p-value of 0.05 (along the post-treatment branches only)^17^; (ii) We used Hyphy v2.3 to fit a model of episodic directional selection (MEDS) to the post-treatment branches pressure using a p-value of 0.05^18^; and (iii) We performed a novel analysis designed to identify positions that changed from an amino acid that is conserved or occurred commonly in PI-naïve individuals to one that is rare in PI-naïve individuals. The rationale for this third analysis derives from the observation that most established HIV-1 drug-resistance mutations involve amino acids that are non-polymorphic in the absence of selective drug pressure.

To accomplish our third analysis, we determined the extent of polymorphism in group M sequences encompassing the entire *gag* from 4,722 individuals and the entire *gp41* from 4,006 individuals in the LANL HIV Sequence Database that we confirmed as PI-naïve through literature review. We then defined a “PI selection index” as log_10_ of the ratio of the prevalence (or site frequency) of the pre-therapy amino acid divided by the prevalence of the post-therapy amino acid. Mutations with a high PI-selection index were defined as changing from a highly conserved or relatively common amino acid variant at a position to an amino acid with a prevalence ten times less common (i.e., selection index ≥1.0).

Gag cleavage site mutations were defined as amino changes between a baseline and follow-up sequence at one of the five residues flanking the matrix (MA)/capsid (CA), CA/SP1, SP1/nucleocapsid (NC), NC/SP2, SP2/p6, and p6/protease boundaries.

To identify mutations that may have resulted from cytotoxic T lymphocyte (CTL) selective pressure, we looked up each mutation in the LANL Immunology HIV Database CTL/CD8+ Epitope Summary file^19^. The second and C-terminal positions within an epitope were referred to as anchoring positions. Anchoring positions at which there were previous reports of CTL escape were considered potential CTL escape mutations.

Because truncated *gp41*-CDs have been shown to reverse the fusion deficit associated with HIV-1 protease inhibition^13, 20, 21^, we noted all *gp41*-CD termination codons and tested whether any observed *gp4*1-CD mutations were predicted to introduce an HIV-1 protease cleavage site. Potential cleavage sites were predicted using the linear support vector machine (LSVM) and orthogonal encoding trained on a large data set based of 4893 octamers of known cleavability^22^. The LSVM output was mapped to the probability of being cleaved using a logistic model, which had been fitted using leave-one-out on the LSVM training data.

## Results

### Description of individuals and samples

Among 41 study subjects, paired sequences before and after PI therapy were available for both *gag* and *gp41* in 11 individuals, for *gag* alone in 13 individuals, and for *gp41* alone in 17 individuals (Table 2). Thirty-eight individuals received ATV/r for a median of 23 months (range: 6 to 81 months) and three individuals received LPV/r for a median of 47 months (range: 5 to 50 months). Overall, the median virus load (VL) before therapy was 4.75 log-copies/ml (interquartile range: 4.15 to 5.1); whereas the median VL following therapy was 4.0 log-copies/ml (interquartile range: 3.45 to 4.4; p < 0.001; paired Wilcoxon Rank Sum test). All but three subjects attained virological suppression at some time during therapy. Ten subjects developed one or more PI (n = 3) or nucleoside RT inhibitor (NRTI) (n = 8) resistance mutations. PI- and/or NRTI-resistance mutations were more likely to develop in the study subjects from KPNC than in the study subjects in the clinical trial A5202 (7/15 vs. 3/26; p = 0.02. Fishers Exact Test).

**Table 2:** Clinical Characteristics of 41 Individuals With gag or gp41 Sequences Before and After Therapy with a First-Line ATV/r or LPV/r-Containing Regimen.

PID	Rx	Group	Age	Sex	CD4	VL_pre_	VL_post_	Rx Months	Achieved VS	PR DRMs	RT DRMs	Genes
8006	ATV/r	KPNC	46	M	620	4	2	81	Y	None	None	*gp41*
14728	ATV/r	KPNC	40	M	158	3.3	3.9	69	Y	None	None	*gp41*
14736	ATV/r	KPNC	26	M	55	3.6	4.3	35	N	50L, 73S	None	*gp41*
18380	ATV/r	KPNC	43	M	215	5.1	4.9	52	N	None	None	*gp41*
24950	ATV/r	KPNC	52	M	590	4.9	3.7	33	Y	None	184V	*gp41*
25082	ATV/r	KPNC	26	M	105	5.1	4.4	18	Y	None	67N	*gp41*
26307	ATV/r	KPNC	38	F	293	5.2	5	77	Y	46I, 50L	184V	*gag, gp41*
38099	ATV/r	KPNC	NA	M	552	4.9	3.6	33	Y	None	None	*gag, gp41*
39143	ATV/r	KPNC	42	M	296	5.7	4.8	49	Y	None	None	*gp41*
39270	ATV/r	KPNC	48	M	224	3.5	2.9	4	Y	None	None	*gp41*
42080	LPV/r	KPNC	36	F	134	4.8	3.1	5	N	43T	None	*gag, gp41*
42654	ATV/r	KPNC	47	M	49	5.4	3.4	18	Y	None	65R, 184V	*gp41*
56120	ATV/r	KPNC	24	M	237	5.5	3.6	41	Y	None	None	*gag, gp41*
56141	LPV/r	KPNC	49	M	424	4.6	2.5	47	Y	None	None	*gp41*
57479	LPV/r	KPNC	35	M	47	2.6	3.5	50	Y	None	184V, 215F	*gp41*
118745	ATV/r	A5202	28	M	301	4.7	3.7	26	Y	None	None	*gag, gp41*
118761	ATV/r	A5202	40	M	40	5.1	4.3	15	Y	None	184V	*gag*
118792	ATV/r	A5202	44	F	187	4.3	3.3	40	Y	None	None	*gag*
118811	ATV/r	A5202	47	M	452	4.1	3.5	11	Y	None	None	*gag*
118823	ATV/r	A5202	23	M	438	4.3	4.4	22	Y	None	None	*gag*
118827	ATV/r	A5202	33	F	434	3.8	4	20	Y	None	None	*gag*
118828	ATV/r	A5202	30	F	112	4.8	4.5	18	Y	None	None	*gag*
118840	ATV/r	A5202	43	M	471	4.7	2.7	19	Y	None	None	*gp41*
118846	ATV/r	A5202	32	M	19	4.6	4.3	12	Y	None	None	*gag, gp41*
118849	ATV/r	A5202	30	M	247	4.6	5.9	41	Y	None	None	*gag*
118853	ATV/r	A5202	37	M	186	3.8	3.6	30	Y	None	None	*gag*
118856	ATV/r	A5202	34	M	543	4.7	4	29	Y	None	None	*gp41*
118860	ATV/r	A5202	46	F	378	5.5	4.3	11	Y	None	None	*gag, gp41*
118886	ATV/r	A5202	61	M	330	4	4.4	26	Y	None	None	*gag*
118899	ATV/r	A5202	50	M	391	5.3	5.9	13	Y	None	None	*gp41*
118903	ATV/r	A5202	36	F	647	3.9	4.1	6	Y	None	None	*gag, gp41*
118910	ATV/r	A5202	34	F	28	6	3.1	23	Y	None	None	*gp41*
118925	ATV/r	A5202	41	M	421	4.8	3.9	14	Y	None	None	*gag*
118935	ATV/r	A5202	24	M	250	5	4.7	26	Y	None	184V	*gag, gp41*
118951	ATV/r	A5202	50	F	177	5	4.3	21	Y	None	None	*gp41*
118954	ATV/r	A5202	32	F	32	4.6	3.2	18	Y	None	None	*gag*
118956	ATV/r	A5202	46	F	10	5.9	3.3	22	Y	None	None	*gag*
118965	ATV/r	A5202	56	M	85	6	4.3	9	Y	None	None	*gp41*
118972	ATV/r	A5202	39	M	404	4.8	4.3	37	Y	None	None	*gag, gp41*
118973	ATV/r	A5202	55	M	24	4.6	6.1	31	Y	None	None	*gag, gp41*
118986	ATV/r	A5202	37	M	NA	NA	NA	NA	NA	None	184V	*gag*

Among 20 NNRTI control subjects, paired sequences before and after NNRTI therapy were available for both *gag* and *gp41* in 13 individuals, for *gag* alone in three individuals, and *gp41* alone in four individuals (Table 3). Seventeen individuals received the NNRTI efavirenz for a median of 17 months (range: 4 to 53); three received the NNRTI nevirapine for a median of four months (range: 4 to 153). Overall, the median VL before therapy was 4.85 log-copies/ml (interquartile range: 4.1 to 5.45); whereas the median VL following therapy was 4.15 log-copies/ml (interquartile range: 3.85 to 4.6; p = 0.001; paired Wilcoxon Rank Sum test). Eleven of 18 evaluable subjects attained virological suppression at some time during therapy. Fifteen of these subjects developed one or more NRTI (n = 6) or NNRTI (n = 15) resistance mutations.

**Table 3:** Clinical Characteristics of 20 Individuals With gag or gp41 Sequences Before and After Therapy with a First-Line NNRTI-Containing Regimen.

PID	NNRTI		Age	Sex	CD4	VL pre	VL post	Rx Months	Achieved VS	RT DRMs	Genes
8349	NVP	KPNC	46	M	164	3.8	4	153	Y	None	*gp41*
9918	EFV	KPNC	38	M	29	5.7	5.5	55	Y	None	*gag, gp41*
21890	EFV	A5202	37	M	426	4.2	3.9	7	Y	None	*gag, gp41*
25036	EFV	KPNC	35	M	43	5.1	4.2	49	Y	65R, 184V, 100I, 103N	*gag, gp41*
35596	EFV	KPNC	40	M	8	5.2	4.7	32	Y	103N, 181C, 190A	*gag, gp41*
37879	EFV	KPNC	45	M	11	5.6	5.2	35	Y	100I, 103N	*gag, gp41*
42036	EFV	KPNC	55	M	35	3.9	4.2	7	N	103N	*gag, gp41*
42183	EFV	KPNC	37	M	442	4.8	5	56	Y	103N	*gag, gp41*
44969	EFV	A5202	26	F	8	4.7	4.5	14	Y	100I, 103N, 184V	*gag*
55928	EFV	KPNC	31	M	206	4.6	4.2	54	Y	103N	*gag, gp41*
57448	NVP	KPNC	28	M	132	3.2	3.1	4	N	181C, 184I	*gp41*
61483	EFV	KPNC	31	M	269	3.9	3.8	11	Y	103N, 190A	*gp41*
61631	NVP	KPNC	31	M	36	5.2	4.1	4	N	65R, 103N, 181C, 184V, 190A	*gp41*
108149	EFV	A5202	44	F	466	4	3.9	7	NA	103N, 190A	*gag*
122034	EFV	A5202	31	M	10	6.1	3.8	6	N	None	*gag, gp41*
214046	EFV	A5202	42	M	19	6.4	4.9	4	N	67N, 103N, 184I	*gag, gp41*
232768	EFV	A5202	38	M	173	4.9	3.7	6	N	41L, 103N, 184I, 190A	*gag*
252392	EFV	A5202	37	M	168	4.9	4	8	NA	103N	*gag, gp41*
252540	EFV	A5202	18	M	242	5.3	4.2	11	Y	103N, 184V	*gag, gp41*
264159	EFV	A5202	28	M	185	6.1	3.6	6	N	103N, 184V	*gag, gp41*

**Figure 1:**
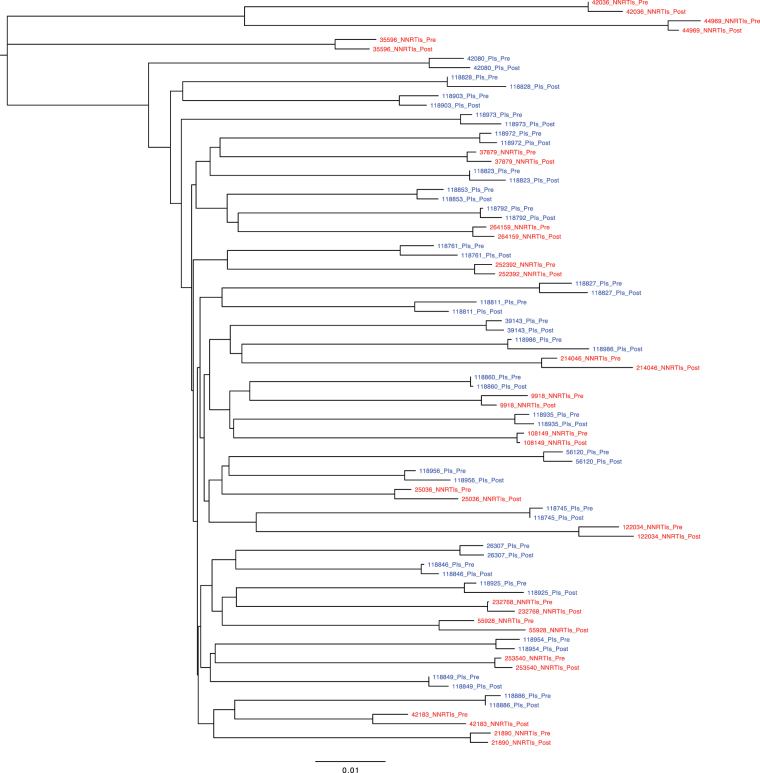
Neighbor joining tree of 40 pairs of *gag* sequences: 24 pairs from individuals before and after therapy with a PI-containing regimen and 16 pairs from individuals before and after therapy with an NNRTI-containing regimen.

**Figure 2:**
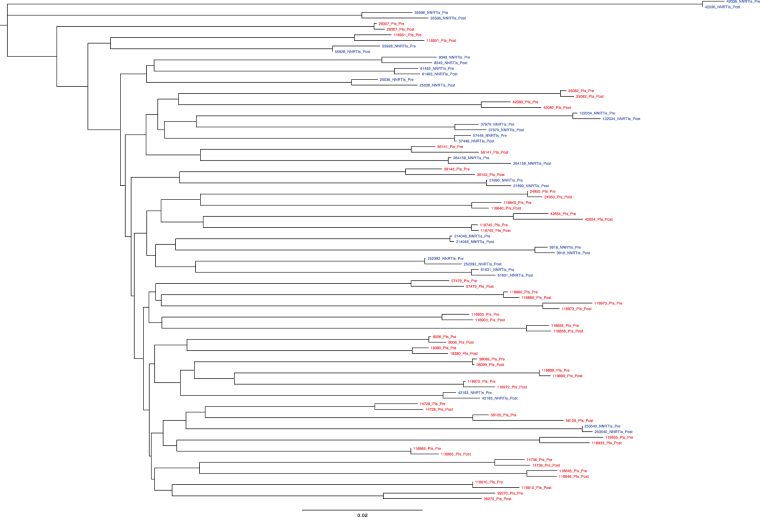
Neighbor joining tree of 45 pairs of *gp41* sequences: 28 pairs from individuals before and after therapy with a PI-containing regimen and 17 pairs from individuals before and after therapy with an NNRTI-containing regimen.

All paired sequences from the 41 study subjects who received PIs and from the 20 control subjects clustered by individual in joint phylogenetic trees (Figs 1 and 2). Each of the study subjects who received PIs had subtype B viruses. Eighteen of the NNRTI control subjects had subtype B sequences; one had sequences belonging to CRF01_AE and another to CRF02_AG.

### Comparison of pre- and post-therapy *gag* sequences

#### Gene-wide analysis

The median proportion of amino acid differences, henceforth referred to as mutations, for the paired *gag* sequences from 24 PI-treated individuals was 1.4% (range: 0.0% to 6.0%) and for the paired *gag* sequences from 16 NNRTI-treated control individuals was 0.8% (range: 0.4% to 4.0%; p = 0.3). In the PI group, there were a total of 175 mutations at 110 positions. In the NNRTI control group, there were a total of 109 mutations at 83 positions.

There was no difference between the median dN/dS ratio in the complete *gag* (PIs 0.25; range: 0 to 0.88 vs. NNRTIs 0.35; range: 0.05 to ∞; p = 0.2), the MA domain (PIs 0.24; range: 0 to ∞ vs. NNRTIs 0.73; range 0 to ∞; p = 0.4), or the C-terminal domain (PIs ∞; range 0.00 to ∞ vs NNRTIs ∞; range 0.00 to ∞; p = 0.6) between paired sequences from PI-treated and control NNRTI-treated individuals. Infinite point estimates arise often when sequences (or regions of sequences) only contain non-synonymous substitutions, but such estimates are highly imprecise.

For the sequences obtained pre- and post PI therapy, RELAX reported a trend for shrinking dN/dS values towards one (neutrality) in the post-therapy compared with the pre-therapy branches (*K* = 0.75; p = 0.07). This non-significant trend, which is consistent with a relaxation of selection pressure, was not observed in NNRTI control group (K = 1.05; p = 0.6).

#### Individual amino acid changes

**Figure 3:**
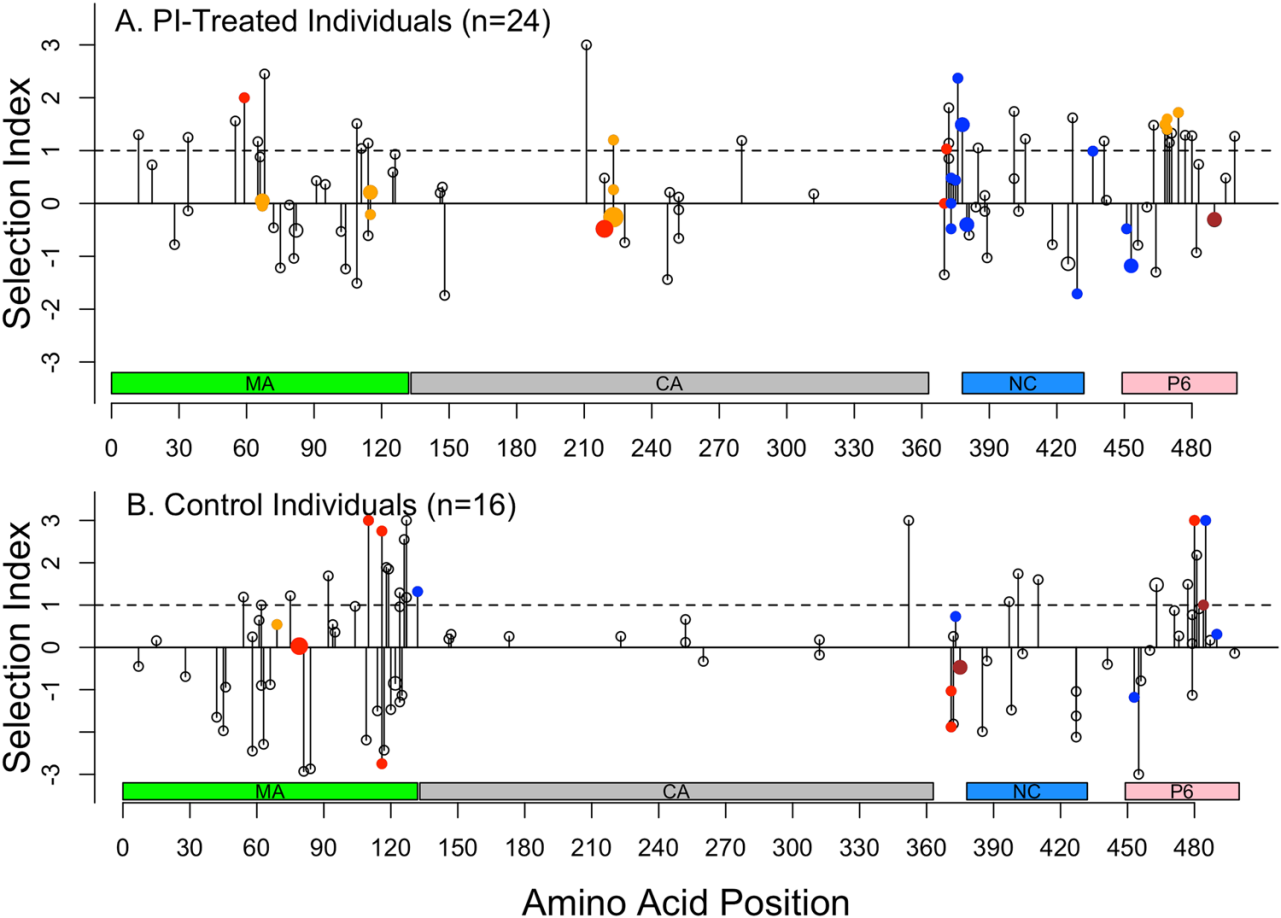
Graphical summary of *gag* sites at which amino acid mutations developed during therapy. Distribution of selection indexes defined as the log_10_ of the ratio of the prevalence of the pre-therapy amino acid divided by the prevalence of the post-therapy amino acid in published group M viruses from ARV-naïve individuals, of protease cleavage site mutations, and of positions displaying evidence for directional selection pressure per MEDS or diversifying selection per FEL in individuals receiving PIs (**A**) or NNRTIs (**B**). The height of each point is the selection index and the size of each point is proportional to the number of occurrences of the mutation. Positions exhibiting diversifying selection are colored orange. Amino acids exhibiting directional selection are colored red (whether or not they also exhibit diversifying selection). Mutations at cleavage sites are colored blue. Mutations at cleavage sites that exhibit diversifying or directional selection are colored brown.

Fig. 3A and B summarize the *gag* sites at which amino acid mutations developed during therapy. The figures show the distribution of selection indexes (defined as log_10_ prevalence of pre-therapy amino acid/prevalence of post-therapy amino acid), cleavage site mutations, and positions with evidence for diversifying selection using FEL or directional selection using MEDS in the 24 PI- and 16 NNRTI-treated individuals.

In the PI group, the amino acids K59M, Q219H, F370Y, and T371N displayed evidence for directional selection and positions 67, 115, 223, 468, 469, and 474 displayed evidence for diversifying selection. Among the mutations with evidence for directional selection, only Q219H occurred in more than one individual. Three individuals receiving PIs developed Q219H and one developed H219Q (Fig. 3A). Position 219 is highly polymorphic with Q and H occurring in 24% and 74% of group M sequences, respectively. Its selection index is therefore log_10_ of 0.24/0.74 = −0.48.

Among the positions with evidence for diversifying selection in the PI group, multiple mutations occurred at positions 67 (A67S in two individuals and S67A in one individual), 115 (A115T in two individuals and T115A in one individual), 223 (V223I in four individuals, V223A in one individual, and I223V in one individual), and 469 (T469A in one individual and I/T469M in one individual). Of mutations at positions exhibiting diversifying selection those at positions 468, 469, and 474 had a selection index above one (Fig. 3A).

In the NNRTI control group, Y79F, K110M, A/N371T, and D480L displayed evidence for directional selection and positions 69, 116, 375, and 484 displayed evidence for diversifying selection. Among the mutations with evidence for directional selection, 371T occurred in two individuals (A371T and N371T) and K110M and D480L had high selection indexes, with K110 occurring in 93% of viruses and D480 occurring in 80% of viruses, while M and L at these positions occurred in 0.02% and 0.06% of group M viruses, respectively (Fig. 3B).

Among the positions with evidence for diversifying selection in the NNRTI control group, positions 375 and 484 are at protease cleavage sites. Mutations at position 116 (A116Q and Q116A) and 375 (A375T in two individuals and A375N in one individual) occurred in more than one individual. Q116A and N484V had selection indexes of 2.7 and 1.0, respectively.

There was no difference in the number of mutations with a high selective index between the PI-treated and control individuals (32/175 vs. 23/109; p = 0.6) (Fig. 3A and B). As the preceding paragraphs indicate there was also no discernible difference in the number of positions with evidence for directional and diversifying selection between the PI and NNRTI groups.

#### Protease cleavage site mutations and PTAP duplications

Twelve individuals in the PI group had 17 cleavage site mutations including 11 SP1/NC, two SP2/P6, and two P6/PR mutations (Fig. 3A). Two of these had a high selective index. I376T at the P2 position of SP1/NC (I and T have prevalences of 80% and 0.3% respectively) occurred in one individual and M378I at the P1’ position of SP1/NC (M and I have prevalences of 94% and 3% respectively) occurred in two individuals. Seven individuals in the NNRTI control group had 9 cleavage site mutations including one MA/CA, four SP1/NC, one SP2/P6, and three P6/PR mutations (Fig. 3A and B). In the PI group, one individual had an insertion of the amino acids PTAP at codon 457.

#### Samples containing PI- or NRTI-resistance mutations

Five of the PI-treated individuals with paired *gag* sequences developed a PI- or NRTI-resistance mutation (Table 2). Supplementary Figure 1 shows the distribution of selection indexes, cleavage site mutations, and positions with evidence for diversifying or directional selection in these five PI-treated individuals and in the 16 NNRTI-treated individuals. These five individuals had six polymorphic cleavage site mutations (P373S, S373P, K380R, K429R, K436R, and R490K), three positions with evidence of diversifying selection and six positions with a selection index ≥1.0. The relatively small numbers of individuals with PI- and/or NRTI-resistance mutations made it difficult to compare the distributions of *gag* mutations in these five individuals with the 19 remaining PI-treated individuals or with the 16 NNRTI-treated individuals.

#### Potential CTL associated mutations

In *gag* sequences from PI-treated individuals, 57 mutations occurred within CTL epitopes including 24 at anchoring positions. At 15 of these 24 positions, there was at least one report of a CTL escape mutation including three positions at which more than one mutation developed in our study: positions 79, 115, 370. These numbers were similar for *gag* mutations in NNRTI-treated individuals, with 58 mutations occurring within CTL epitopes including 22 at anchoring positions. At 12 of these 22 positions, there was at least one report of a CTL escape mutation. Of note, the positions with the most mutations in the PI group, codons 219 (n = 4) and 223 (n = 6) in CA, were not CTL-escape mutations but rather mutations that have been reported to covary with several known CTL escape mutations^23^.

#### Nucleotide ambiguities

In the PI group, 63 (36%) of the 175 mutations were changes from a pre-therapy mixture encoding two amino acids to one of the possible resolutions of the mixture in the post-therapy sequence. Consistent with the observation that intra-host diversity decreased following therapy, was the finding that the median proportion of nucleotide ambiguities pre-PI therapy was significantly greater than the proportion post-PI therapy: 0.43% (IQR:0.07% to 0.80%) vs 0.04 (IQR:0% to 0.55%; p = 0.02 paired Wilcoxon Rank Sum test).

A similar but less marked reduction in nucleotide and amino acid mixtures was observed in the NNRTI group, in which 17 (16%) of the 109 mutations were from a pre-therapy mixture encoding two amino acids to one of the resolutions post-therapy. Similarly, the pre-therapy sequences had a higher proportion of nucleotide ambiguities compared with the post-therapy sequences: median 0.13% (IQR: 0.07% to 0.13%) vs. 0% (IQR: 0% to 0.1%; p = 0.04 paired Wilcoxon Rank Sum test).

### Comparison of pre- and post-therapy *gp41* sequences

#### Gene-wide analysis

The median proportion of mutations for the paired *gp41* sequences from 28 PI-treated individuals was 1.6% (range: 0.0% to 6.2%) and for the paired *gp41* sequences from 17 NNRTI-treated control individuals was 1.3% (range: 0.0% to 3.9%; p = 0.95). In the PI group, there were a total of 153 mutations at 91 positions. In the NNRTI control group, there were a total of 90 mutations at 62 positions.

There was no difference between the median dN/dS ratio in the complete *gp41* (PIs 0.42; range: 0 to ∞ vs. NNRTIs 0.54; range: 0.18 to ∞; p = 0.3) or *gp41* CD domain (PIs 0.59; range: 0 to ∞ vs. NNRTIs 0.45; range 0 to ∞; p = 0.8) between paired sequences from PI and NNRTI treated individuals. For the sequences obtained pre- and post PI therapy, RELAX reported a trend for shrinking dN/dS values towards one (neutrality) in the post-therapy compared with the pre-therapy branches (*K* = 0.70; p = 0.06). This non-significant trend, which is consistent with a relaxation of selection pressure, was not inferred in NNRTI control group (*K* = 0.87; p = 0.63).

#### Individual amino acid changes

**Figure 4:**
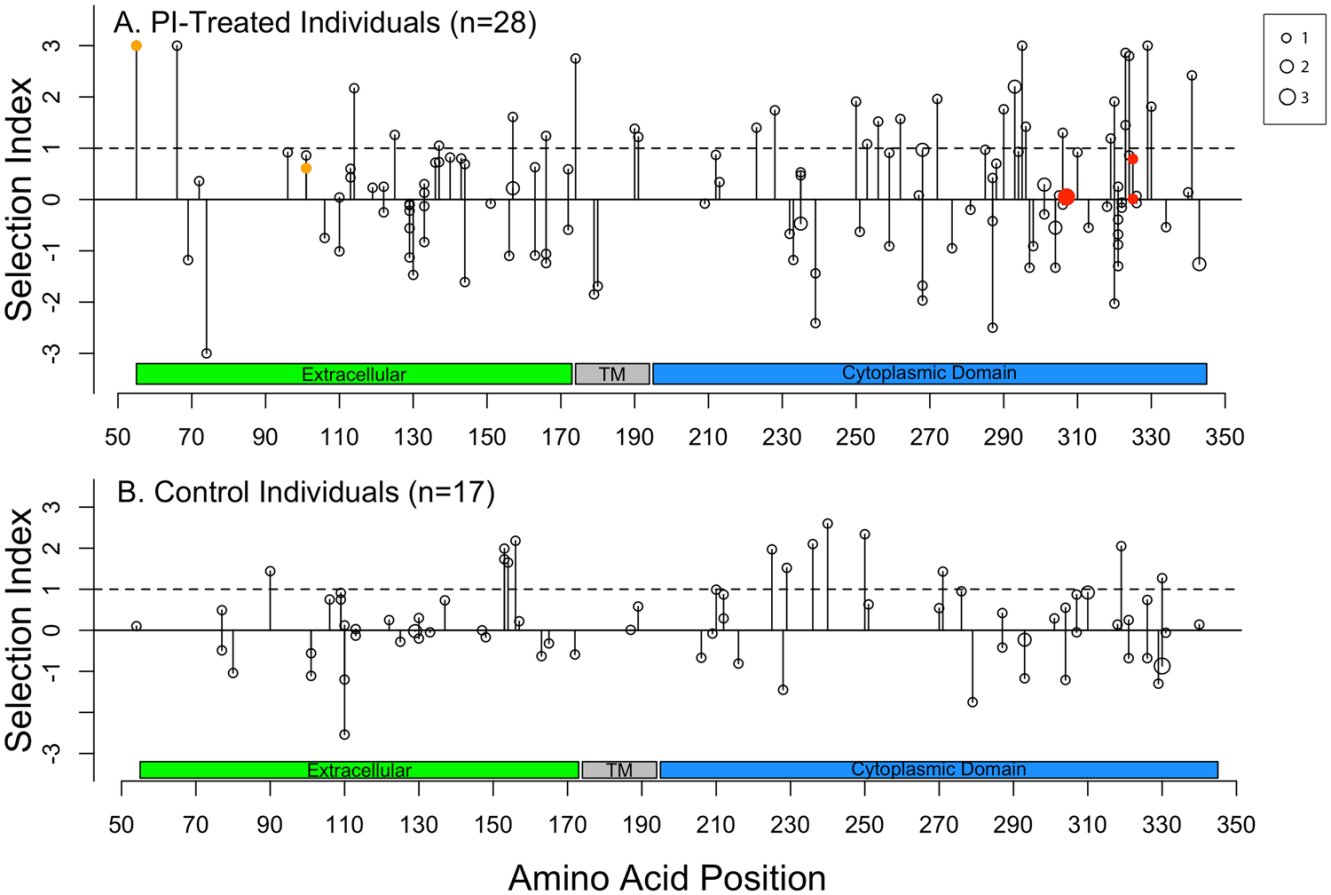
Graphical summary of *gp41* sites at which amino acid mutations developed during therapy. Distribution of selection indexes defined as the log_10_ of the ratio of the prevalence of the pre-therapy amino acid divided by the prevalence of the post-therapy amino acid in published group M viruses from ARV-naïve individuals and of positions displaying evidence for directional selection pressure per MEDS or diversifying selection per FEL in individuals receiving PIs (**A**) or NNRTIs (**B**). The height of each point is the selection index and the size of each point is proportional to the number of occurrences of the mutation. Positions exhibiting diversifying selection are colored orange. Amino acids exhibiting directional selection are colored red (whether or not they also exhibit diversifying selection). Abbreviation: transmembrane (TM).

Figure 4A and B show the distribution of selection indexes and positions with evidence for diversifying selection using FEL or directional selection using MEDS. In the PI group, the amino acids T307I and I/L325F displayed evidence for directional selection by MEDS and positions 55 and 101 displayed evidence for diversifying selection by FEL. Of the mutations with evidence for directional selection, T307I occurred in three individuals, and I325F and L325F each occurred in one individual. Of note, these mutations had selective indexes below <1.0. Mutations at positions 55 and 101 occurred just once. One of these, L55W had a selective index of 3.3 as the prevalence of L in ARV-naïve individuals is 99.7%, whereas the prevalence of W is 0.3%. In the NNRTI control group, no mutations displayed evidence for directional selection and just position 24 displayed evidence for diversifying selection.

There was no difference in the number of mutations with a high selective index between the PI-treated and control individuals (30/153 vs. 13/90; p = 0.4). As the preceding paragraphs indicate there was also no discernible difference in the number of positions with evidence for directional and diversifying selection between the PI and NNRTI groups.

#### Introduction of potential cleavage site mutations and premature termination codons in gp41-CD

Among the 28 individuals in the PI group, 522 unique octamers occurred in sequences after but not before treatment. A Kolmogorov-Smirnov test on their estimated probabilities for being cleaved by HIV-1 protease indicated no statistically significant difference in cleavage susceptibility between those octamers present only before treatment and those present only after treatment. A similar test on the 322 unique octamers present after treatment in the NNRTI group also demonstrated no significant difference in cleavage susceptibility. Among the 5% of octamer mutations resulting in the largest increase in cleavage susceptibility, 34 (71%) of 48 were from the PI group and 14 (29%) of 48 were from the NNRTI group – ratios consistent with larger number of individuals in the PI group. One virus from a PI-treated individual developed a premature termination codon as part of a mixed virus population at *gp41* position 286 (TGG→TRG; W286W*).

#### Samples containing PI- or NRTI-resistance mutations

Eight of the PI-treated individuals with paired *gp41* sequences developed a PI- or NRTI-resistance mutation (Table 2). Supplementary Figure 2 shows the distribution of selection indexes and positions with evidence for diversifying or directional selection in these eight PI-treated individuals and in the 16 NNRTI-treated individuals. These eight individuals had 13 mutations with a selection index ≥1.0, two mutations at positions with evidence of diversifying selection, and three mutations with evidence of directional selection. The relatively small numbers of individuals with PI- and/or NRTI-resistance mutations made it difficult to compare the distributions of *gp41* mutations in these eight individuals with the 20 remaining PI-treated individuals or with the 17 NNRTI-treated individuals. There also appeared to be no difference in the probability that a cleavage site was introduced was introduced into *gp41*-CD in the eight individuals with PI- or NRTI-resistance mutations compared with the 20 PI-treated individuals without such mutations.

#### Potential CTL associated mutations

In *gp41* sequences from PI-treated individuals, 62 mutations occurred within CTL epitopes including 16 at anchoring positions. At 4 of these 16 positions, there was at least one report of a CTL escape mutation. These numbers were similar for NNRTI-treated individuals with 42 mutations occurring within CTL epitopes including 14 at anchoring positions. At 4 of these 14 positions, there was at least one report of a CTL escape mutation.

#### Nucleotide ambiguities

In the PI group, 17 (11%) of the 153 mutations were changes from a pre-therapy mixture encoding two amino acids to one of the resolutions post-therapy. The median proportion of nucleotide ambiguities before PI therapy was also higher than the proportion of nucleotide ambiguities following therapy: 0.11% (IQR: 0% to 0.23%) vs 0.04 (IQR: 0% to 0.11%; p = 0.08 paired Wilcoxon Rank Sum test). No consistent pattern of changes at codons containing nucleotide ambiguities was observed in *gp41* in the NNRTI group.

## Discussion

Shortly after the introduction of PIs, several C-terminal NC/p6 and SP2/p6 *gag* cleavage site mutations were found to compensate for the reduced fitness associated with primary PI-resistance protease mutations^24–29^. In the ensuing years, additional cleavage site mutations as well as several C-terminal non-cleavage site *gag* mutations were shown to either develop in viruses from patients receiving PIs, to reduce PI susceptibility in site-directed mutants, or to occur more commonly in virus sequences containing PI-resistance protease mutations^12, 30–34^. More recently, several studies used pseudotyped viruses to show that genetic loci in the N-terminal MA *gag* domain^35–39^ and in *gp41*-CD^13^ can reduce PI susceptibility even in the absence of PI-resistance protease mutations. Nonetheless, no *gag* changes have been consistently observed in individuals with VF while receiving a PI-containing regimen^40^, and there have been no studies of *gp41*-CD sequences in viruses from patients before and after receiving a PI-containing regimen.

Our study is the first to analyze entire *gag* and *gp41* sequences before and after PI/r therapy in which *gag* and *gp41* changes are compared to those occurring in a control group with VF on an NNRTI-containing regimen. We found in both the PI- and NNRTI-treated groups that many amino acid mutations developed in *gag* in *gp41*. However, in neither gene, was there a discernable difference in the overall numbers of mutations, number of mutations displaying evidence of diversifying or directional selection, or numbers of mutations with a high selection index between the two treatment groups. Moreover, many of the observed mutations, particularly in *gag*, may reflect a reduction in quasispecies diversity following the bottleneck of treatment-induced virological suppression (rather than selection by an ARV class) as evidenced by the decrease in the proportion of nucleotide ambiguities in post-therapy sequences in both the PI and NNRTI groups.

Several potentially PI-associated *gag* mutations occurred in the PI group including 17 cleavage site mutations, one PTAP duplication, and four mutations at codon 219 – a highly polymorphic position involved in cyclophilin A incorporation^12, 30, 32^. Two of the cleavage site mutations – I376T in one individual and M378I in two individuals – are of interest because they each had a high selective index and have not been previously described. However, individuals in the NNRTI-control group also developed multiple cleavage site mutations. The positions with the most mutations in the PI group (positions 219 and 223) are highly polymorphic sites that have also been reported to increase viral fitness particularly in viruses with *gag* CTL-escape mutations^23^. Several mutations in both groups also occurred at CTL sites.

The concept that *gp41*-CD contain determinants of PI susceptibility is supported by mechanistic studies showing that protease inhibition interferes with *gp41*-mediated fusion^20, 21, 41^. Moreover, several studies have shown that *gp41*-CD truncation can reverse the fusion deficit associated with HIV-1 protease inhibition^13, 20, 21^. However, our analysis of the observed *gp41*-CD mutations in the PI group did not provide evidence for the frequent introduction of premature termination codons or novel *gp41*-CD cleavage sites that might provide virus escape from a PI-induced fusion defect.

A significant limitation to this study is the likelihood that nonadherence contributed to VF in some of the individuals in our cohort. Although all but three individuals receiving PIs were adherent enough to attain virological suppression on therapy, only ten individuals developed one or more established PI or NRTI-resistance mutations. The fact that nonadherence is a major contributor to VF and that nonadherence to PI/r therapy may be less likely to select for resistance than nonadherence to other ARV classes^6–8^ underscores the need for even larger datasets than ours to identify PI/r-selected mutations.

Within the protease, primary drug-resistance mutations cause a fitness loss such that secondary mutations in both protease and gag usually emerge to increase virus fitness^12, 42^. In contrast to primary drug-resistance mutations, these fitness-enhancing mutations are often polymorphic, pre-existing within an individual’s virus population before the start of therapy. Unless the selection pressure on individual sites is extremely high, statistical methods for detecting directional selection require larger numbers of patients than those we studied, or stronger selective effects^18, 43^. Moreover, it may be that the genotypic determinants of reduced susceptibility in *gag* and/or *gp41* are not mediated by interactions between specific residues, but rather by nonspecific steric effects that allow HIV-1 to complete its replication cycle despite having an immature virion. The inter-molecular interfaces involved in the interactions between protease and the *gag* protein product are large, making it plausible that the impact of any single individual intermolecular interaction is small^44^.

Despite the many studies in which a considerable number of *gag* mutations have been shown to either emerge during PI therapy or to reduce PI susceptibility, it remains uncertain which, if any, of these mutations cause clinically significant reductions in PI susceptibility in the absence of PI resistance protease mutations^12, 45^. Our study shows that if *gag* and/or *gp41* encode PI-resistance mutations, they are unlikely to be confined to consistent mutations at a handful of sites. Their identification will therefore require larger numbers of paired sequences from individuals with VF on a PI/r-containing regimen, or more sensitive methodology.

### Data Availability

The sequence data in this study are available in GenBank under the accession numbers listed in the Methods. The sequences can be linked to data from the individual from whom the samples were obtained using the anonymous PIDs included in Tables 2 and 3.

### Ethics Approval and Informed Consent

As stated in the Method section, this study was approved by the Institutional Review Boards (IRBs) of Stanford University, KPNC, and the NIH ACTG. The NIH ACTG samples were obtained from study subjects who provided informed consent for participation in a clinical trial. The KPNC samples were remnant plasma samples obtained following routine genotypic antiviral drug resistance testing. The Stanford University and KPNC IRB approvals included a waiver of consent to use these samples for this study.

## Electronic supplementary material

Supplementary Information
