# Supplementary material for: Evolution of gag and gp41 in Patients Receiving Ritonavir-Boosted Protease Inhibitors
Source: Sci Rep. 2017 Sep 14;7:11559. doi: 10.1038/s41598-017-11893-8 (PMC5599673; doi:10.1038/s41598-017-11893-8)

SUPPLEMENARY INFORMATION

Evolution of *gag* and *gp41* in Patients Receiving Ritonavir-Boosted Protease Inhibitors

By

Justen Manasa, Ph.D.[1]

Vici Varghese, Ph.D.[1]

Sergei L. Kosakovsky Pond, Ph.D.[2]

Soo-Yon Rhee, Ph.D.[1]

Philip L. Tzou, B.S.[1]

W. Jeffrey Fessel, M.D[3]

Karen S. Jang, B.S.[1]

Elizabeth White, Ph.D.[1]

Thorsteinn Rögnvaldsson, Ph.D.[4]

David A. Katzenstein, M.D.[1]

Robert W. Shafer, M.D.[1]

[1]Division of Infectious Diseases, Department of Medicine Stanford University, Stanford, CA; [2]Department of Biology, Temple University, Philadelphia, PA.; [3]Department of Internal Medicine, Kaiser Permanente Northern California, San Francisco Medical Center, San Francisco, CA' [4]School of Information Technology, Halmstad University, Halmstad, Sweden.

Corresponding author: Robert W. Shafer, M.D., Division of Infectious Diseases, Department of Medicine, Stanford University, Stanford, CA.; email: rshafer@stanford.edu; 650-725-2946

Supplementary Figure 1

Graphical summary of *gag* sites at which amino acid mutations developed during therapy. Distribution of selection indexes defined as the $\log_{10}$ of the ratio of the prevalence of the pre-therapy amino acid divided by the prevalence of the post-therapy amino acid in published group M viruses from ARV-naïve individuals, of protease cleavage site mutations, and of positions displaying evidence for directional selection pressure per MEDS or diversifying selection per FEL in the five PI-treated individuals with a PI- or NRTI-resistance mutation (A) or in the individuals receiving NNRTIs (B). The height of each point is the selection index and the size of each point is proportional to the number of occurrences of the mutation. Positions exhibiting diversifying selection are colored orange. Amino acids exhibiting directional selection are colored red (whether or not they also exhibit diversifying selection). Mutations at cleavage sites are colored blue. Mutations at cleavage sites that exhibit diversifying or directional selection are colored brown.

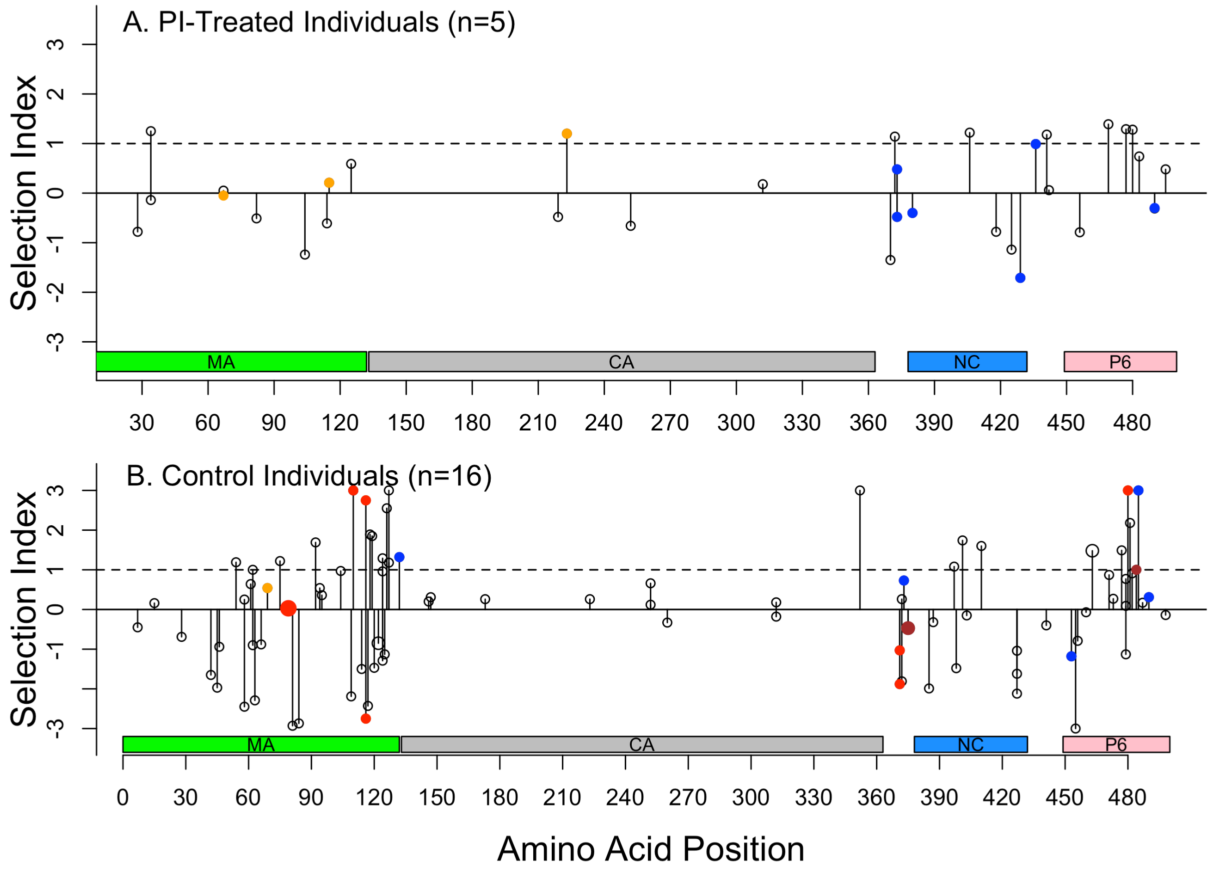

Supplementary Figure 2

Graphical summary of *gp41* sites at which amino acid mutations developed during therapy. Distribution of selection indexes defined as the $\log_{10}$ of the ratio of the prevalence of the pre-therapy amino acid divided by the prevalence of the post-therapy amino acid in published group M viruses from ARV-naïve individuals and of positions displaying evidence for directional selection pressure per MEDS or diversifying selection per FEL in the eight PI-treated individuals with a PI- or NNRTI-resistance mutation (A) or in the individuals receiving NNRTIs (B). The height of each point is the selection index and the size of each point is proportional to the number of occurrences of the mutation. Positions exhibiting diversifying selection are colored orange. Amino acids exhibiting directional selection are colored red (whether or not they also exhibit diversifying selection). Abbreviation: transmembrane (TM).

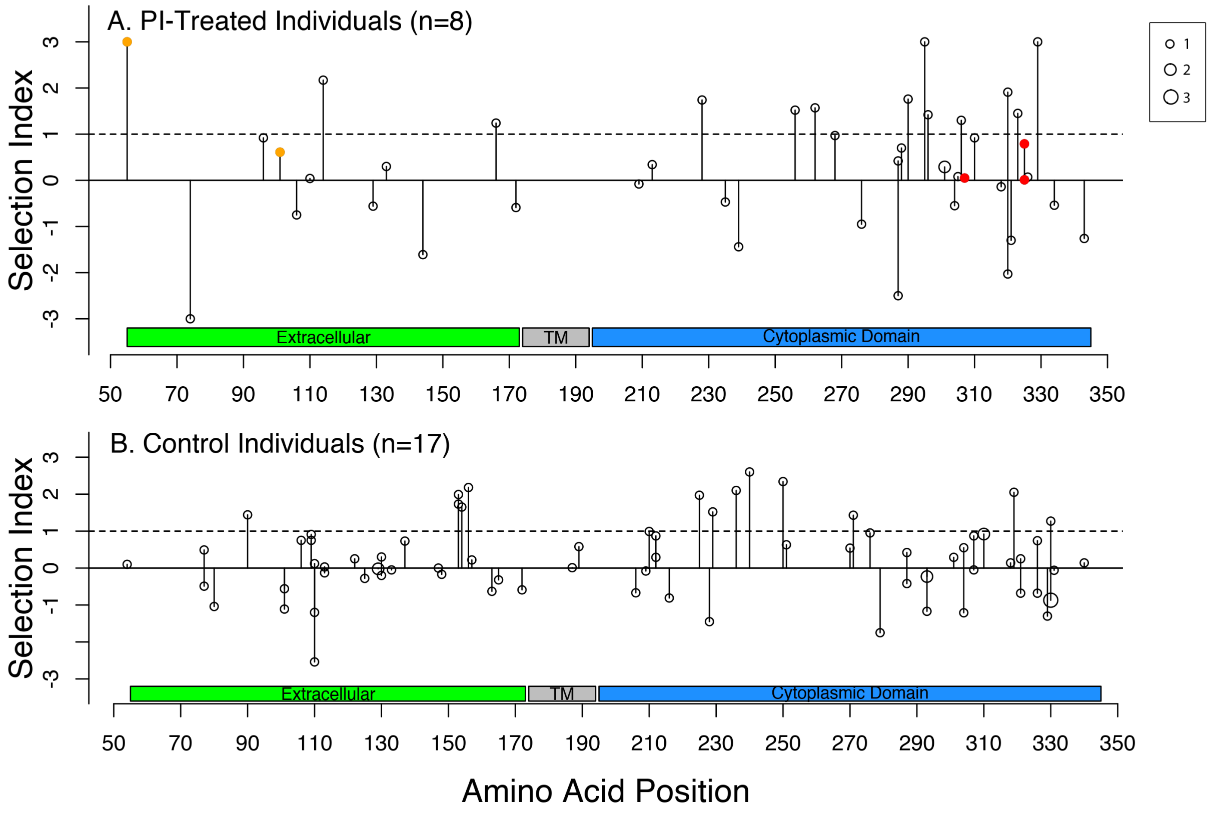

Supplement: Supplementary file 1 — Supplementary Information [file 41598_2017_11893_MOESM1_ESM.pdf]
